# The Effects of COVID-19 Among the Elderly Population: A Case for Closing the Digital Divide

**DOI:** 10.3389/fpsyt.2020.577427

**Published:** 2020-11-12

**Authors:** Gabrielle Martins Van Jaarsveld

**Affiliations:** Department of Psychology, Education & Child Studies, Erasmus University Rotterdam, Rotterdam, Netherlands

**Keywords:** COVID-19, pandemic, digital divide, digital literacy, elderly

## Abstract

The COVID-19 pandemic has had huge effects on the daily lives of most individuals in the first half of 2020. Widespread lockdown and preventative measures have isolated individuals, affected the world economy, and limited access to physical and mental healthcare. While these measures may be necessary to minimize the spread of the virus, the negative physical, psychological, and social effects are evident. In response, technology has been adapted to try and mitigate these effects, offering individuals digital alternatives to many of the day-to-day activities which can no longer be completed normally. However, the elderly population, which has been worst affected by both the virus, and the lockdown measures, has seen the least benefits from these digital solutions. The age based digital divide describes a longstanding inequality in the access to, and skills to make use of, new technology. While this problem is not new, during the COVID-19 pandemic it has created a large portion of the population suffering from the negative effects of the crisis, and unable to make use of many of the digital measures put in place to help. This paper aims to explore the increased negative effects the digital divide is having in the elderly population during the COVID-19 pandemic. It also aims to highlight the need for increased attention and resources to go toward improving digital literacy in the elderly, and the need to put in place measures to offer immediate solutions during the COVID-19 crisis, and solutions to close the digital divide for good in the long-term.

## Introduction

As the COVID-19 crisis evolves, the widespread effects of both the virus and the preventative measures being taken to protect the population are becoming clearer. At the time of writing this paper, the total number of confirmed cases of the virus has surpassed 34 million, and the number of deaths is over 1 million, and increasing daily (1). The economic consequences of this crisis have been immense, and researchers have suggested that the effect on world economies is likely to be felt for years (2–5). However, the COVID-19 crisis has brought with it a whole selection of other problems, including those not directly related to the virus, but to the lockdown measures which have been put in place across the globe. While the lockdown may be necessary to contain the virus, its effects, ranging from physical to psychological have already been noted. Early studies have suggested that the psychological effects of this crisis and the prolonged lockdown includes increased stress, anxiety and depression (6–11). Researchers have also warned to brace for a possible spike in suicide rates in the months following the crisis (12). In many regions the pandemic has caused difficulty accessing healthcare resources for non-COVID related problems (13), resulting in higher risk of poor outcomes for those suffering from other diseases (14). The disruption of workplaces, exercise routines, and widely imposed social isolation are all likely to have a large effect on the well-being of the population going forward. While there will not be a group of the population untouched by this crisis, the elderly population is likely to face the worst effects. Initial reports have shown that ~80% of the deaths due to COVID-19 occur in those over the age of 65 (15). Since the virus has largely affected the elderly, lockdown measures for older individuals have been stricter, and may need to be extended in some countries (16, 17). This means that the elderly will be most impacted by the side effects that follow in the coming months (18).

While the changes and restrictions in daily life are noticeable and immense in many cases, digital tools and resources have been highlighted as possible means of mitigating the worst of the negative consequences. Social isolation has well-documented negative effects on well-being in individuals of all ages, but the effect has been shown to be magnified in older adults (19, 20). Social isolation often results in loneliness, which is a factor significantly associated with depression in elderly adults (21). Loneliness, isolation, and depression have all been shown to predict worse disease outcomes in older populations (22). Furthermore, depression and other mental health issues are linked to higher mortality rates in general, in those over 65 years old (23). The use of technology to continue to stay in touch with family, friends and loved ones has become an important way to combat these negative effects associated with prolonged loneliness and isolation. Virtual socializing and online events have become commonplace and have gone a long way to keeping people from being completely isolated while in lockdown (24, 25). The ability to remain in contact with friends and family via online video chat tools may also offer individuals more socializing opportunities to avoid loneliness. Online education has also become the new normal in many places, as schools and universities turn to online classes to keep student education on track (26). Furthermore, as individuals have more flexible schedules, or more free time during the lockdown, there has been a significant increase in the number of people making use of personal learning and development tools like language learning apps (27). Healthcare has also turned to digital solutions, and making both mental and physical healthcare available online has become more common and has been fairly successful in helping mitigate the negative effects of reduced healthcare access (28–34).

While technology may have gone a long way to mitigate negative effects of the crisis in the general population, the situation is more complicated in the elderly population. Access to, and ability to proficiently use technology is much lower in older populations than in younger adults (35, 36). This uneven distribution of technological access and skill is known as the digital divide, or the gray digital divide, and researchers have suggested it has continued to increase as the rate of technological innovation speeds up (37). This results in a paradoxical situation, in which the population most affected by the lockdown is also the population least helped by the digital tools aiming to mitigate the negative effects. This paper aims to highlight the negative effects of COVID-19 in the elderly population and explore how uneven access and proficiency in technology is contributing to increased negative outcomes within this population. This paper will end by making practical suggestions for how this digital divide can, and should, be addressed going forward.

## The Effects of COVID-19 on the Elderly Population

Although it is currently unclear what the full extent of the effects of this pandemic will be, its negative impact on psychological well-being has become very evident. Early studies have already reported an increase in anxiety, and depression in the general population, especially those facing extended lockdowns (38, 39). These effects are magnified in the elderly population due largely to stricter lockdowns, higher threat of illness, and loss of social support (40). Prior studies have also reported that even outside of crisis times, the elderly population have relatively high rates of depressive symptoms (41, 42), which is troubling in the face of evidence that those suffering from pre-existing mental health conditions have been most affected by the negative psychological consequences of lockdowns (7). While increased mental health problems in the general population may already be a cause for concern, these concerns go beyond psychological well-being in the elderly. Studies have shown that depression in the elderly is linked the subsequent cognitive decline, and risk of Alzheimer's Disease (43, 44). This means that while many societies now face the immediate threat of increasing mental health concerns, the long-term effects could be devastating, as depression and stress result in the older generation facing hastened cognitive decline, and increased rates of Alzheimer's Disease. This problem will likely be even further worsened by the physical limitations put on the movement of individuals outside their homes, resulting in less exercise opportunities for many individuals. Several studies have shown that exercise, even in light to moderate doses and intensities, can have a significant positive effect on cognitive function in the elderly, especially in those with cognitive impairments, or neuropsychiatric disorders (45–49). Looking at this prior research, loss of socialization, increased mental strain and general mental health problems, and decreased exercise, could have substantial negative effects on the elderly population. Although the lockdowns may be temporary, these effects are likely to be long lasting, and could pose significant risks to the quality of life of the elderly population in the coming years.

However, the changes many countries have seen come into place since the start of the COVID-19 pandemic extend far beyond loss of socialization, and increased depression. Lockdowns have resulted in a significant shift in the functioning of day-to-day life: the world has gone digital. As hospitals have filled with COVID-19 patients, access to regular healthcare for non-COVID related disorders has been interrupted (50). Those who do not seek care for non-COVID related disorders may be at higher risk of illness and fatality during this period (51). This risk is likely to disproportionately affect the elderly, who have higher rates of health problems than younger populations and are more likely to be encouraged to avoid areas where they could contract the disease. In response to this problem, there has been a significant shift in healthcare into the digital world. Telehealth, or the act of providing healthcare digitally, and remotely, has become commonplace in many countries (28, 30, 32, 34). However, this shift has had fewer positive effects in the elderly than other populations. A recent study showed that about 40% of elderly individuals were unprepared to use telehealth resources, predominantly due to lack of skills to effectively make use of the technology (52). This has been further shown during the pandemic, as the group with the highest adoption of telemedicine use has been those aged 20–44, despite the fact that the elderly population generally have the highest yearly number of doctor and hospital visits (53, 54). Although there have been some recent efforts to create virtual geriatric clinics to support the elderly during the pandemic, research has shown these have had varying success, and have been met with a variety of problems related to difficulties with technology use (55). Therefore, despite being the group most in need of telehealth solutions, the elderly community has benefited from their implementation the least.

This shift into the digital realm extends beyond just the healthcare sector. Online access to COVID-19 related news, education, grocery delivery services, group socialization, and many more services have become commonplace. The world has adapted to try and make up for the loss of access to everyday resources, and in many areas, and for many people, this has been fairly effective (56–59). However, one group likely to benefit the least from these digital alternatives are the elderly population, who have significantly lower rates of internet usage and acceptance than other age groups (60, 61). This results in a worrying paradox: the population most negatively affected by the COVID-19 pandemic, are also the least likely to be able to access the resources put in place to mitigate the effects. This paradox can largely be attributed to the poor digital literacy skills found amongst the elderly population compared to younger groups, most commonly described as the digital divide.

## The Digital Divide

The digital divide is a term originally used to describe the gap in access to new technology which exists between different groups of people (62). Early research on this topic mostly focused on the differences in technological accessibility within poorer communities or countries (63–65) or the growing gender based digital divide (66–68). However, as technology has advanced and become more engrained in our daily lives, the case of the digital divide has become more complex. An article by (62) developed a model which suggested four different levels of technological access which the digital divide has an effect on. These levels included (1) Motivational Access, (2) Material Access, (3) Skills Access, (4) Usage Access. This makes an important distinction between a digital divide which exists on the basis of uneven material access to technology, a digital divide based on uneven motivation to use technology, and a digital divide based on uneven distribution of technological skills and ability to make use of technology.

In Western countries today, access to the internet, and use of technology in general is extremely high. In European countries more than 82.5% of the population uses the internet, and 86.5% of households have internet access (69). However, these numbers fail to capture a specific aspect of the digital divide: that which exists in the elderly population in Western countries. Statistics examining the use of, and access to, the internet collect less data from older participants, due to practical limitations, and often apply an upper age limit to their sample (35). This results in data which represents access and use of technology in the general adult population but fails to capture the significant gap in access among the elderly. Studies which examined the difference in technology access and use in the elderly have found that age significantly predicts not only lower access to technology, but also within technology users, less frequent and varied usage (35, 36). This results in a troubling conclusion: not only does the elderly population in Western countries have less access to technology than younger adults, but even those with access have less digital skills, and make more limited use of the technology they do have. This conclusion mirrors results from studies on digital literacy which have found that the elderly often have lower levels of skilled, competent use of technology in their daily lives (70, 71).

There are therefore several reasons for the existence of the so-called gray divide in elderly populations. Although fundamental access to technology may be a problem among some groups, especially those in poorer communities, rates of access to internet is generally quite high, especially in Western countries, and studies have shown that cost or ability to access technology only play a small role in the reason for lack of usage in older individuals (35). Instead, research suggests that the main determinants of this divide are low motivational access, and a general skills deficit (35). A recent study showed that elderly individuals who reported disliking technology mainly attributed this to the belief that it was inconvenient, or that the costs outweighed the benefits (72) The task of closing the digital divide therefore becomes an issue of not only improving elderly access to technology, and offering skills training so they can develop digital skills, but also implementing programs to increase the elderly population's motivation to use technology, and better understand the benefits it can offer. In the case of a lack of motivational access, community-based interventions may be especially beneficial, as they would allow for widespread targeting of the elderly, with the aim of encouraging transfer of motivation within the community as more individuals adopted technology usage.

The problem of the digital divide among the elderly is not new and has been a point of increasing scrutiny as technology has become a larger part of day-to-day life. However, while some studies and programs have attempted to explore possible solutions, little headway has been made on a large scale (73–75). Many studies on the topic of technology usage in the elderly focus on the design of technology and software which the elderly are more easily able to use, which has resulted in a variety of hardware and software design suggestions to tailor technology to the needs of elderly users (76–78). This research has shown that the elderly are more likely to own outdated technology than their younger counterparts, and can benefit from the design of simple user interfaces, and cost-friendly technology alternatives (79, 80). While this is a very important step which will lay the basis for how technology can be used by the elderly, focusing on community wide programs to improve digital access, motivation, and skills should be the next focus. The COVID-19 pandemic has had a huge impact on the global community, and the long-term side-effects are likely to be felt for years to come. This pandemic has also shifted the way individuals are using technology and has highlighted the importance of closing the digital divide amongst the elderly, to try and minimize the negative effects this crisis will have on an already highly affected portion of the population.

## Mitigating the Effects of the Digital Divide

While the digital divide in the elderly population is certainly not a new problem, the COVID-19 pandemic has made it clear that some immediate action needs to be taken to address it. In the short-term, there is a need to ensure that digital solutions to lockdown problems are also accessible to older populations. As of 2015, about 8.5% of the world population was aged 65 or older, and this number is growing every year (81). This is not a small group of people, and during the COVID-19 pandemic it is essential that society remains aware of the challenges they are facing and takes measures to mitigate them. Encouraging the use of digital solutions in elderly groups is necessary, and governments and care homes should take measures to ensure the elderly population is aware of the resources available online during this pandemic. Raising awareness of the resources which can be accessed and making them available to less technologically savvy older individuals could have large benefits. Online socializing events catering to older individuals would allow for social contact, without any risks of COVID-19 infection. The introduction of online exercise programs geared toward homebound older individuals could offer simple workout routines to reduce the physical risks of decreased exercise. While short-term measures are unlikely to reach all older individuals, especially those with minimal material access to technology, they could help maximize the usefulness of digital tools in older individuals without current knowledge of their availability.

While the short-term goals of tackling the digital divide should focus on minimizing the harmful effects of the COVID-19 pandemic, the long-term goals should focus on taking meaningful steps to close the digital divide between older and younger populations. Governments should be taking steps to put in place programs which increase access to technology and offer older individuals the opportunity to learn how to use them. Care homes and community centers should also take the opportunity to implement digital literacy programs for older individuals. These measures will need to take into account the differences in reasons for the digital divide which exist across various socio-economic and gender groups. Older individuals in poorer communities may face a larger problem from a lack of material access to technology, and in those communities an initial focus supporting the purchase and upkeep of technological resources for elderly groups may be required. However, in wealthier communities, the problem is more likely to rest on a lack of motivation to use technology and a lack of digital skills. Therefore, initiatives targeting those communities will more likely need to start with programs aimed at increasing motivation for technology use, and digital skills training. Differences in education level and literacy levels in the general community should also be taken into account to ensure that the correct programs can be implemented to target the underlying reasons for the digital divide.

Prior studies have shown that digital literacy programs for older individuals can be very effective and have long-term effects on their digital skills (74, 82). Furthermore, they have shown that programs and applications developed specifically for the elderly can result in a significant improvement in confidence and interest in using technology (83). Most of these programs involved digital skills training, which in turn resulted in increased self-efficacy and motivation to continue using technology. Research on the development and implementation of digital literacy training programs for the elderly is not lacking, merely the motivation to implement these programs on a large scale. Studies show that perceived ease of use, and perceived usefulness are both important aspects predicting use of technology among older populations (84). Both of these factors are can be targeted by information campaigns and community-based programs to help the elderly understand how technology can help them in their day-to-day lives. Increasing affordable access to technology, motivating usage, and improving overall digital skills must all form part of a complete campaign to decrease the uneven usage of technology. Given the current display of the harmful effects of the digital divide, and the fact that reliance on the internet, and technology in general, is likely to increase in the coming years, it is overdue, but more necessary than ever to take action and start to make changes that will contribute to the closing of the digital divide.

## Conclusion

As the COVID-19 pandemic has progressed, the unforeseen side-effects have started to make themselves known. As lockdowns across the world change the day-to-day life of billions of people, the world has had to adapt to the changes. The shift to a focus on digital tools has been successful in minimizing many of the problems faced during the pandemic, and many individuals have continued to socialize, study, work and access healthcare via digital tools. However, the elderly population, who have historically faced a large inequality in access to, and ability to make use of technology, has not seen the same benefits as many other younger groups. The elderly population has been hit with some of the worst effects of the pandemic, with harsher lockdown measures, and increased risks of mental and physical health problems, and the digital divide has seen that the effects of these measures have not been minimized. There is a definite need for action, both in the short and long-term to minimize the negative effects the digital divide has during this pandemic, and to act to close the divide in the long term. Action by governments to increase access to technology and implement digital literacy programs in elderly populations is absolutely necessary, especially going forward into an increasingly digital future. While actions now many not be able to completely shield the elderly from the negative effects of the pandemic, they could minimize them, and ensure that going forward this issue is given the attention and resources it needs to finally close the age based digital divide.

## Data Availability Statement

The original contributions presented in the study are included in the article/supplementary material, further inquiries can be directed to the corresponding author.

## Author Contributions

GM wrote, reviewed, and revised the manuscript.

## Conflict of Interest

The author declares that the research was conducted in the absence of any commercial or financial relationships that could be construed as a potential conflict of interest.
